# Teams in Small Organizations: Conceptual, Methodological, and Practical Considerations

**DOI:** 10.3389/fpsyg.2021.530291

**Published:** 2021-03-18

**Authors:** Roni Reiter-Palmon, Victoria Kennel, Joseph A. Allen

**Affiliations:** ^1^Department of Psychology, University of Nebraska Omaha, Omaha, NE, United States; ^2^Department of Allied Health Professions Education, Research, and Practice, University of Nebraska Medical Center, Omaha, NE, United States; ^3^Department of Family and Preventive Medicine, University of Utah, Salt Lake City, UT, United States

**Keywords:** teams, methodology, teamwork, team composition, team effectiveness, team processes

## Abstract

Research on teams and teamwork has flourished in the last few decades. Much of what we know about teams and teamwork comes from research using short-term student teams in the lab, teams in larger organizations, and, more recently, teams in rather unique and extreme environments. The context in which teams operate influences team composition, processes, and effectiveness. Small organizations are an understudied and often overlooked context that presents a rich opportunity to augment our understanding of teams and team dynamics. In this paper, we discuss how teams and multi-team systems in small organizations may differ from those found in larger organizations. Many of these differences present both methodological and practical challenges to studying team composition and processes in small complex organizational settings. We advocate for applying and accepting new and less widely used methodological approaches to advance our understanding of the science of teams and teamwork in such contexts.

## Introduction

Teams are a prevalent force in today’s organizations (Hackman, 2011). Teams are often formed to accomplish tasks that no single individual could reasonably accomplish within the time specified (Kozlowski and Ilgen, 2006). Kozlowski and Ilgen (2006, p. 79) defined teams as “(a) two or more individuals who (b) socially interact (face-to-face or increasingly, virtually); (c) possess one or more common goals; (d) are brought together to perform organizationally relevant tasks; (e) exhibit interdependence concerning workflow, goals, and outcomes; (f) have different roles and responsibilities; and (g) are together embedded in an encompassing organizational system, with boundaries and linkages to the broader system context and task environment.” Therefore, teams are deployed in a variety of organizations for multiple purposes (Forsyth, 2018).

As work demands become increasingly complex and teams in organizations begin to collaborate to achieve goals, an intricate system of interdependent efforts emerges among teams, often referred to as a multi-team system (DeChurch and Zaccaro, 2010). Multi-team systems are defined as teams of teams collaborating together, often in response to challenging environmental contingencies, towards fulfilling collective, shared goals (Marks et al., 2001). The multi-team system exists in large organizations and small ones, in dangerously complex environments, and in environments that are not inherently dangerous but are no less complex (Shuffler et al., 2015).

The context in which teams and multi-team systems operate is an increasingly important concept of interest in the study of real-world teams. Context may be defined as the “situational opportunities and constraints that affect occurrence and meaning of organizational behavior as well as functional relationships between variables” (Johns, 2006, p. 386). Johns asserted that researchers too readily overlook the impact of context on results and inferences made about the phenomena studied, and this likely contributes to variation in research findings across studies. Teams researchers have dedicated greater attention to context and its effects on team performance (Golden et al., 2018). However, context can also influence issues such as study design and the methods necessary to effectively understand team phenomena, including those methods used to study real teams as they operate in unique contexts (Tannenbaum et al., 2012). For example, Bell et al. (2018) offered a detailed rationale regarding why existing team literature and research approaches were inappropriate for research on teams working under extreme conditions. Their proposed methodology enabled meaningful study and actionable research of small samples of teams working in such contexts.

**Figure 1:**
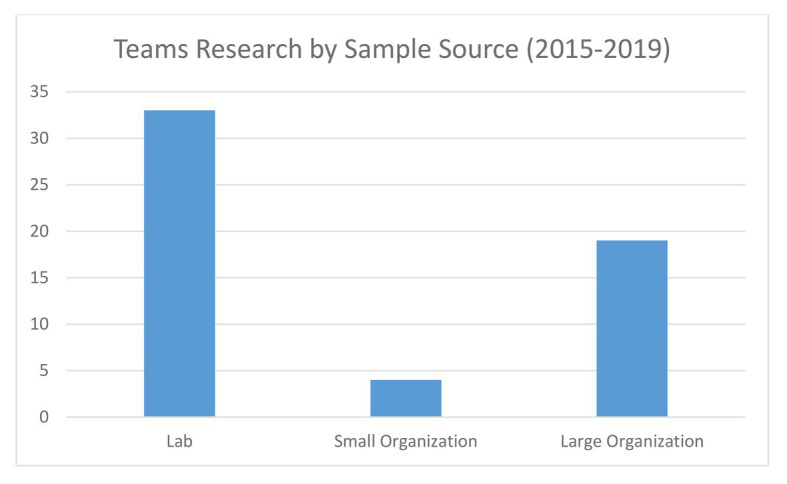
Teams research articles by sample source type comprised of 2015–2019 literature.

While much can be learned regarding team composition, processes, and effectiveness from teams in the laboratory and large organizations, an important type of environment or context has been excluded from the study. Many employees are not working in large organizations. For example, in the United States, according to the Small Business Administration (2018), small businesses (*n* < 500 employees) are responsible for 47.5% of private-sector employment, and are responsible for 66% of new jobs created between 2000 and 2017. Furthermore, while the definition of a small business is less than 500 employees, many organizations are much smaller than that. Much of the research on teams in Psychology and Management, however, has occurred using either lab studies where data can be usually easily gathered and conditions are well-controlled, or in larger organizations where researchers can find a sufficient number of teams to allow for traditional statistical analyses, and, hopefully, generalizable results. For example, a search of recent literature (2015–2019) on teams and team performance from a variety of Psychology and Management journals (i.e., *Journal of Applied Psychology, Academy of Management Journal, Small Group Research, Journal of Organizational Behavior, Journal of Management, Group and Organization Management, and Group Processes and Intergroup Relations*), found a total of 58 articles published on the topic and only four of those included samples from small organizations, representing less than 10% of the publications (see Figure 1; Table 1). Thus, small organizations and businesses pose a unique context that is underrepresented in existing teams literature and has not been fully explored. This is especially troubling as small organizations represent a substantial subset of organizations and employ a significant portion of the workforce. As a result of the limited research, we are unclear on how the context of small organizations relates to and affects our current understanding of team composition and processes. This presents an opportunity for additional conceptual development and a discussion ofmethodological and measurement challenges and opportunities for the study of teams and teamwork.

**Table 1:** Summary of team research conducted in small organizations.

Study authors	Year	Type of organization(s)	Number of organizations	Constructs evaluated
Hu, J., Erdogan, B., Jiang, K., Bauer, T. N., and Liu, S.	2018	Information and technology	11	Leader humility, power distance, information sharing, psychological safety, creativity
Dietz, B., van Knippenberg, D., Hirst, G., and Restubog, S. L. D.	2015	Sales in IT, hospitality, financial services	12	Goal orientation, team identification, performance
Hajro, A., Gibson, C. B., and Pudelko, M.	2017	Chemical, oil, banking, transportation, electronic, construction, consulting, technology, retail	11	Qualitative study: diversity climate, knowledge exchange, effectiveness
Herdman, Yang, and Arthur	2017	Manufacturing, technology, hospitality	7	Leader-member exchange, Leader-leader exchange, teamwork behavior, team effectiveness

The purpose of this article is 2-fold. First, we discuss how teams in small organizations may differ from teams in larger organizations. We use elements of the Input-Process-Output (IPO; Forsyth, 2018) model and the Critical Conditions for Teamwork Heuristic (Salas et al., 2015) as guiding frameworks for this discussion. The IPO model is a classic systems model of teamwork suggesting that inputs lead to processes that then lead to outcomes. Inputs represent the composition of the team and contextual factors such as resources available and culture. Processes reflect activities that members of the team engage in to address the demands of the team. Outputs refer to the team’s performance. Given that this model is used across most teams research, it provided the initial guide to the structure of this paper. The Critical Conditions for Teamwork Heuristic (Salas et al., 2015) is a practical framework intended to consolidate major findings from the teamwork literature and provide guidelines for teamwork that are useful in practice. This heuristic describes three influencing conditions – context, composition, and culture – and six core processes and emergent states – cooperation, coordination, cognition, conflict, coaching, and communication. We focus our discussion on the influencing conditions (i.e., the inputs) – namely, context and composition – and core processes and emergent states (i.e., the processes) – cognition, communication, coordination, and coaching – that we believe are most strongly affected by the unique contextual elements of working in small organizations. We use this terminology to organize our discussion within the paper.

Second, we address methodological challenges that may arise from studying teams in this context, where methodology and research design that has been used for studying teams in larger organizations and in the laboratory may not be sufficient or appropriate. The goal of this paper is not to provide a review of all possible methodological considerations for studying teams in this context. We intend to start a discussion on how small organizations provide a different context than that of larger organizations. Therefore traditional methodologies may not be sufficient for the study of teams in small organizations. We then propose methodological and measurement strategies that expand upon traditional team data collection and methodological practices to open the door for new rules and ways to conceptualize and operationalize key elements of team composition and processes.

## Teams in Small Organizations: Conceptual Considerations

### Influencing Conditions

Broadly speaking, influencing conditions represent the factors that impact team processes and emergent states (Salas et al., 2015). Such factors include the broader context in which teams operate, the team’s composition, and culture. In the following section, we discuss how these influencing conditions – specifically, context and composition – may help us understand teams’ unique characteristics in small organizations.

#### Context

The context in which teams operate influences the way in which teams are formed and how team members work together (Salas et al., 2015). Small organizations are an understudied and often overlooked context that presents a rich opportunity to augment our understanding of teams and team dynamics. For this paper, small organizations may include small businesses and small to medium-sized enterprises (Organization for Economic Co-operation and Development, 2021), a term used more globally. The two most obvious and fundamental distinguishing characteristics of small organizations are size, defined by the number of employees within the organization, and annual revenue (Small Business Administration, 2018).

An early challenge to understanding the context of small organizations is that it was “assumed that their ‘simple’ characteristics and the existence of ‘evident’ solutions to their problems did not provide for substantive research questions” (Raymond, 1985, p. 37). Thus, organizational theory applied in their study was based primarily on the study of large-sized organizations (Dandridge, 1979), and small businesses were treated as “merely smaller versions of large businesses” (Keats and Bracker, 1988, p. 41). Early efforts to expand management theory to the study of small businesses included analyzing the task environment, the configuration of the organization, and characteristics of management (d’Amboise and Muldowney, 1988). Beyond size and revenue, small organizations may differ in characteristics from larger organizations in terms of organizational structure, ownership and management, resource availability, human resource and development functions, and systems and processes (Welsh and White, 1981; d’Amboise and Muldowney, 1988; Hill, 2001; Cardon and Stevens, 2004; Wong and Aspinwall, 2004). These characteristics shape the context in which individuals and teams organize and operate within the small organization setting.

#### Composition

Research on team composition explores team members’ various attributes and how the combination(s) of such attributes affects team processes, emergent states, and team outcomes (Mathieu et al., 2014). Such attributes may exist at the surface-level as they are readily detectable or easily accessed by others, such as sex, race, age, and role, while others such as personality traits, expertise, and values may exist at a deeper-level (Bell et al., 2018). A variety of reviews and meta-analyses have demonstrated that team composition factors relate to team outcomes and performance (e.g., Devine and Philips, 2001; Peeters et al., 2006; Bell, 2007; Bell et al., 2011); thus, appropriate team composition is of importance to work teams across a variety of organizations.

##### Team Diversity

The first issue faced by teams in small organizations is that of achieving desired levels of team diversity. Demographic diversity may be limited in these smaller organizations – either due to geographic location (e.g., rural) or hiring practices (e.g., hiring within family or friend network; Anderson and Reeb, 2004). Also, small organizations may experience difficulty in creating functionally diverse teams. First, many small organizations choose to contract out some functions that may be included in larger organizations. For example, functions such as accounting, human resources, or marketing may be outsourced to a specialized company with the requisite expertise. As such, it is more likely that these functions will not be represented when teams are created. Second, in smaller organizations, members may need to fill multiple roles and have expertise in multiple areas. Therefore, functional diversity may need to be evaluated and measured differently in teams in small organizations.

##### Multiple Team Membership

A second issue arises for teams in small organizations as individuals may be called upon to serve on multiple teams, and be required to “wear many hats” (O’Leary et al., 2011). While multiple team membership can occur in larger organizations, we suggest that this is more likely to influence a larger number or proportion of people in smaller organizations as the number of individuals that can be tapped for participation in a team is smaller. Further, in addition to quantitative differences (more people affected) there are qualitative differences in working across multiple teams within a small organizational context. In large organizations, membership in multiple teams results in time fragmentation and less social support, leading to increased job demands (Pluut et al., 2014). In small organizations, this may not be the case, as individuals are more likely to know and have previous interactions with multiple team members, outside of the specific team. Moreover, as more team members are likely to be in the same position of being part of multiple teams, they are more likely to be aware of the difficulties associated with the fragmented work, and potentially be more understanding and supportive. This in turn may result in a different dynamic than what we expect to see in larger organizations. However, increased job demands may remain an issue, or perhaps may be more salient, when the same sets of individuals are asked to participate in many different teams, simply because no one else is available to serve on any given team.

##### Fluid Team Membership

A third issue that teams in small organizations must address is that of making the most of dynamic team membership. Many models of teams do not explicitly indicate whether team membership is stable and constant but may take this view implicitly (Kozlowski and Bell, 2003; Reiter-Palmon et al., 2017). Further, much of the empirical work conducted on teams fails to include a dynamic view, including dynamic team membership (Cronin, 2015). Recent conceptualizations of team membership change suggest each change functions as a specific “event” which may affect team functioning in different ways, depending on the novelty, disruptiveness, and critical nature of the change (Trainer et al., 2020). From a methodological perspective, this stability of membership allows one to conclude that the relationships and effects under investigation are not confounded by any changes in the team’s composition and membership. However, a more contemporary and, perhaps a more realistic approach suggests that stable and clearly defined membership on teams is an increasingly rare phenomenon in practice (Wageman et al., 2012). That is, in many real-world circumstances, team membership may not be a stable phenomenon, and treating it as such creates barriers to the study of real-world teams.

Over time, dynamic team membership is likely in small organizations due to frequency of multiple team membership and increased job demands of serving on multiple teams described earlier. Because of this issue, team membership may naturally ebb and flow over time. Fluid team membership results from team membership instability where members “come and go” during the team’s life cycle (Bushe and Chu, 2011). This is often the result of factors such as changes in desired skill sets for various team needs and stages of work, work scheduling and personnel availability, and turnover (Bushe and Chu, 2011). From a team composition perspective, fluid team membership may be conceptualized in ways similar to that of multiple team membership and may include relevant aspects such as the length of time one member serves on a team, during what specific stages of team performance, and for what purpose. Fluid team membership poses several challenges to effective team performance, such as knowledge loss, lack of shared mental models, issues with team commitment, and low cohesion (Bushe and Chu, 2011). However, the benefits of multiple team membership among teams in small organizations may help to buffer the effects of some of these challenges.

### Core Processes and Emergent States

In addition to influencing conditions, team processes and emergent states are often evaluated in team research and may differ dramatically between smaller and larger organizations. Team processes reflect how team members interact, combine efforts, and coordinate work to address task work (Kozlowski and Ilgen, 2006), and are affected by the context in which teams operate (Kozlowski, 2015). Such processes are often conceptualized as phenomena that emerge from individuals’ interactions within teams (Kozlowski and Klein, 2000), but are often treated as static in research (Kozlowski and Chao, 2012). Alternatively, they can be treated as phenomena that emerge and yet vary or fluctuate over time (Kozlowski, 2015). Examples of team processes include cognitive processes and structures such as team mental models and transactive memory, and team learning; interpersonal, motivational, and affective processes such as team cohesion, efficacy, conflict; and, team action and behavioral processes such as coordination, communication, and regulation (Kozlowski and Ilgen, 2006; Lehmann-Willenbrock and Allen, 2018). We present our discussion of core processes and emergent states as they align with some of the relevant critical considerations of teamwork: cognition, communication, coordination, and coaching (Salas et al., 2015). Our discussion of these factors acknowledges the impact that the influencing conditions discussed in the prior section may have on the emergence and manifestation of these team processes and states.

#### Cognition

Team cognition considerations include developing a shared understanding among team members, which may include knowledge of team member roles and responsibilities, team members respective knowledge and abilities, team goals, and team norms (Salas et al., 2015). A recent meta-analysis summarized the importance of team cognition for effective team performance and characterized the circumstances in which this team process mattered most (Niler et al., 2020). The concepts of team mental models and transactive memory “refer to cognitive structures or knowledge representations that enable team members to organize and acquire information necessary to anticipate and execute actions” (Kozlowski and Ilgen, 2006, p. 83). Klimoski and Mohammed (1994) proposed that team mental models represent a shared understanding and organization of beliefs or knowledge that relate to the team’s task environment; in other words, the knowledge and information team members hold in common. Studies of team mental models indicate their positive effects on team performance (e.g., Mathieu et al., 2000), and interventions such as cross-training (Marks et al., 2002) and leadership pre-briefs (Marks et al., 2000) are reported to support the development of team mental models. Transactive memory refers to the collection of individual memory systems within the team that combines what team members know with the shared understanding of who possesses the knowledge; in other words, knowing who knows what within the team (Wegner, 1995).

Given the likelihood of “multiple hats” and multiple team membership within teams in smaller organizations, as described earlier, it is also likely that shared team cognition may develop more easily for teams in small organizations and would be easier to maintain over time. Shared knowledge of who knows what, who does what, and how to do the work should help improve coordination and action among team members. Thus, teams in smaller organizations may have an easier time developing aspects of team cognition, and reap the benefits of shared cognition on team performance. However, when turnover occurs, it may be more difficult for the individual replacing the original team member to assimilate and incorporate the shared mental models and transactive memory, as a result of serving in multiple roles and teams.

#### Communication

Communication across team members as well as across teams (multi-team systems) and leadership has been identified as one of the most important factors that lead to team success. Salas et al. (2015, p. 607) define communication in teams as a “reciprocal process of team members’ sending and receiving information that forms and re-forms a team’s attitudes, behaviors, and cognitions.” Effective team communication has been shown to improve team effectiveness, creativity and innovation, and overall performance (Mesmer-Magnus and DeChruch, 2009) and reduce errors (Allen et al., 2018). In addition, communication has been found to influence other relevant teamwork characteristics such as shared mental model, trust and conflict. Effective and positive communication has been found to facilitate the development of shared mental models and trust, and reduce conflict (Reiter-Palmon and Murugavel, 2018).

In addition to internal communication with the team, communication with other teams, organizational leaders, and other entities is important. Team boundary spanning refers to a team’s effort to establish and manage interactions with others external to their team (and sometimes organization) that may enhance the team in meeting performance goals and others’ performance goals (Van Osch and Steinfield, 2016). Specifically, teams may recognize their own limitations in aspects such as knowledge, expertise, and access to resources. Upon doing so, they may engage in an environmental scanning effort that includes other teams within and external to their organization that may have some of the information, expertise, resources or other items of need. When they discover these potential connections and opportunities, an effort to “boundary span” may arise that leverages network connections across the boundary or that require the forging of new connections (Marrone, 2010). In large organizations, these boundary-spanning efforts may occur in house and involve completely independent or co-dependent groups. They likely also include external teams in other related organization that may mutually benefit from the combined efforts (Van Osch and Steinfield, 2016).

In contrast, small organizations may have multiple teams, with compositional overlap between the teams. From a boundary-spanning perspective, this may actually present opportunities for natural networking and connecting between teams that would not naturally exist in more independent teams in larger organizations. However, it may also limit the amount of resources and unique ideas available by the constrained number of participants in the overlapping teams. Further, the artificial or actual distance between organizations may create unique barriers to boundary spanning for both small and large organizations. We emphasize it as a challenge for small organizations, perhaps more so than larger ones, because the access to resources to shrink that distance *via* actual travel or technology is potentially limited more so in small organizations. This team overlap and membership in multiple teams may result in natural boundary-spanning; however, this may be pose difficulties in measurement. For example, when an individual belongs to multiple teams, do we assume that boundary spanning occurs just because of shared membership? In fact, researchers may want to be explicit about measuring actual activities of boundary-spanning so that they are aware of the actual (instead of implicit assumption) boundary-spanning and specific activities. In larger organizations, boundary-spanning is typically conducted by a limited number of individuals, usually those in leadership positions. However, in small organizations, because boundary-spanning occurs more organically and potentially with more individuals involved, care must be taken to identify the specific aspects of boundary-spanning individuals engage in.

#### Coordination

Coordination represents “the process of interaction that integrates a collective set of interdependent tasks” (Okhuysen and Bechky, 2009, p. 463) and helps teams transform their resources into outcomes (Salas et al., 2015). In their review of the coordination literature, Okhuysen and Bechky (2009) suggested several mechanisms that organizations and teams use to facilitate coordination. Several of these have specific implications for work conducted by teams in small complex organizations. One important aspect in which team members coordinate is by defining responsibility for tasks. Role definition in teams in small organizations is typically less clearly defined than in larger organizations. In addition, increased familiarity may make formal roles less meaningful and informal roles may emerge more quickly. The methodological challenge here is capturing both the formal and informal roles, as well as when roles are clearly defined, and identifying how these roles inform and affect task delegation and responsibility. Another important aspect of coordination is creating and developing a common perspective (Okhuysen and Bechky, 2009). This collective perspective is especially necessary for MTSs as individual teams within the MTS must coordinate their activities to achieve the MTS’s greater goal(s) (Mathieu et al., 2000).

#### Coaching

Leadership has long been recognized as an important factor influencing team and organizational success (Morgeson et al., 2010). However, much of the research conducted on leadership focuses on leadership of specific and static teams (either in the lab or field) or on high level top management teams of large organizations (Bass and Bass, 2008). While much of what we know about leadership theories, types, and styles will likely hold in these small organizations, some notable differences need to be acknowledged and potentially may change how leaders operate, what makes them effective, and influence our methodology.

One important difference between smaller and larger organizations has to do with levels of management (Dalton et al., 1980). Because of their size, small organizations will likely have fewer layers of management between the lowest level employee and the CEO or head of the organization. Fewer layers of management can create an environment where top leadership is much more involved and aware of front line employees’ day-to-day activities. Leaders may be more knowledgeable of and can more directly influence organizational processes and the actions of various groups of employees. Further, fewer levels of management may result in perceptions that leaders are more approachable due to less power distance across levels of leadership. This indicates a need for methodologies and measures that adequately capture the effects of leadership dynamics on team activities and outcomes.

## Teams in Small Organizations: Methodological Considerations and Proposed Solutions

In the following sections, we present several methodological challenges as they emerged from our discussion above on critical considerations for teamwork as they apply to teams in small organizations. We organized our summary of these challenges related to the relevant influencing conditions and team processes and emergent states as described in the prior section. We follow this discussion with potential methodological solutions.

### Influencing Conditions

In this section we discuss unique methodological challenges for measuring and evaluating influencing conditions, specifically, team composition, in small organizations. For each of these challenges, we draw on our theoretical discussion outlined in the previous sections and then offer some potential solutions. Table 2 provides a summary of our discussion of challenges and proposed solutions.

**Table 2:** Team composition methodological considerations for teams in small organizations.

Team composition factor	Composition factor defined	Special considerations for study in small organizations	Methodological and evaluation considerations	Potential solutions
Team diversity Functional diversity	Distributional differences among team members with respect to a given attribute (Harrison and Klein, 2007) Functional knowledge and expertise contribution by members of a team (Bunderson and Sutcliffe, 2002)	Availability of necessary expertise “in-house” vs. external to the organization Team members with “multiple hats” who hold multiple areas of expertise	Potential for limited variance to explore diversity with existing means such as separation, variety, and disparity Role stability within and across teams, and the fulfillment of these roles at any time, emerges as a key team composition consideration Diversity metrics need to consider “interdisciplinary individuals” as they offer complex indications of expertise that may fill multiple specific roles Fluidity of team membership and participation result in frequent changes in team members with knowledge, skills, and experience needed to fulfill team roles	Conceptualize and operationalize team composition as collection of team roles necessary for effective team performance, and the subsequent skill variety necessary to fulfill such roles Engage individuals, team members, and managers to identify roles, knowledge, and skills essential for the team over the life cycle of the team Document analysis of team records and experience sampling may provide valuable qualitative data to help inform the dynamic nature of team membership and contribution
Dynamic membership Fluid membership Multiple team membership	Teams with unstable membership where members “come and go” during the team’s life cycle (Bushe and Chu, 2011) Teams with members who participate in multiple teams at one time (O’Leary et al., 2011)	Limited numbers of staff and expertise may result in team members participating in multiple teams Multiple “hats” in roles on multiple teams may result in significant loss of necessary expertise when team members lack engagement with or leave the team Relative familiarity of team members may lessen the impact of fluidity and multiple membership on team processes		

#### Team Diversity

Methodological and measurement challenges relevant to aspects of team composition and practical challenges, emerge as they relate to conceptualizing and operationalizing team diversity and the functional expertise needed to accomplish team goals. Although these challenges are certainly not unique, in smaller organizations, they are driven primarily by factors specific to the context of small organizations. For example, the limited number of employees available in each organization to participate in teams, recruitment and personnel selection constraints, resource restrictions that affect employment and developmental opportunities, and so forth, all make team composition dynamics an issue.

The distributional differences among team members with respect to a given attribute defines the concept of team diversity (Harrison and Klein, 2007). Such attributes may reflect differences in social category (e.g., gender and ethnicity), knowledge or skills (e.g., functional knowledge and expertise), values or beliefs, personality, organizational or community status (e.g., tenure), and social network ties (Mannix and Neale, 2005). Several published reviews of the literature demonstrate the mixed effects of various types of team diversity on team performance (Bell, 2007; Horwitz and Horwitz, 2007; Bell et al., 2011). Conceptual models of team diversity include those proposed by Harrison and Klein (2007) *via* separation (i.e., lateral differences among members on an attribute), disparity (i.e., vertical differences among members), and variety (i.e., categorical differences among members on an attribute). Of particular interest are the effects of team diversity as defined by functional knowledge and expertise contribution by team members (Bunderson and Sutcliffe, 2002), as this deep-level construct has demonstrated effects on team performance (Bell, 2007; Bell et al., 2011). Importantly, to maximize the benefits of functional diversity under the information-processing tradition (Hinsz et al., 1997), the variety conceptualization (Harrison and Klein, 2007) of functional backgrounds and expertise is required among team members to solve complex problems in small workplace settings.

Under the informational diversity-cognitive resources perspective (Curşeu et al., 2007), team members from different areas can draw from their functional information or resource pools to create a broader understanding of the issues involved. However, there may be several practical challenges to creating the desired composition of team members. First, due to facility size and budget limitations, some facilities may simply lack team members with the expertise necessary to fulfill the necessary roles on these teams. If feasible, some facilities may utilize contracted services to address such a gap. However, individuals working under these circumstances may or may not be readily available to participate consistently within an ongoing team within the facility. This limits teams’ opportunities to realize the benefits of specific functional areas of expertise needed for team success. From a methodological perspective, it is unclear how to categorize such an individual as well as the team, as the availability of this needed expertise area may have fluctuated over time, or, is non-existent. A dynamic perspective that considers such fluctuations in composition and expertise and does not assume stability of membership is needed because team membership may vary by performance episodes. Both degree of variability or stability as well as more qualitative differences (which expertise, when in the timeline of the life of the team changes occur) must be taken into account. As a result, team researchers must not assume a degree of stability and should seek methods that enable them to document actual team composition more carefully and frequently. In-depth document analysis (Bowen, 2009) of records such as reports, meeting minutes, project management tools, or experience sampling methods (Hektner et al., 2007) such as daily diaries can inform our understanding of team composition dynamics at a granular level. Longitudinal research designs also allow for the study of team composition over time. Analytically, utilizing multiple observations over time would require considering the nested nature of the data, for example, using hierarchical linear modeling or similar analytic approaches.

Second, some team members may have multiple areas of expertise in the organization. This would ensure that specific expertise is available to the team, but creates challenges in how that particular team members’ expertise is recognized in practice and operationalized in the research. From a functional diversity and team role perspective, a team member with multiple areas of expertise could be considered an “interdisciplinary individual” as this single individual fulfills various functional expertise and role needs that might have otherwise been filled by more than one person. From a methodological perspective, it is unclear how to classify this team member when operationalizing functional diversity and role. If we count the person as representing multiple domains, our accounting of which disciplines are represented is accurate, but it may artificially inflate the team size. Alternatively, we can count the person as representing one main discipline, but then we may falsely assume that a specific area of expertise was missing from the team. However, in practice, we cannot be certain whether the person indeed will represent multiple perspectives or only one perspective, and under what circumstances (e.g., what does one do when the respective expertise areas may have conflicting perspectives on an issue). Qualitative data such as task analyses and team meetings observations may help shed some light on how these interdisciplinary individuals approach their position and role in the team. This data could further inform the appropriate way to classify such team members.

New directions in the conceptualization and operationalization of team composition factors have been explored recently in the literature (Mathieu et al., 2014; Bell et al., 2018). These approaches build upon the traditional compositional models by which teams have profiles where the contributions of team members are weighted equally, to those compilational models that emphasize the relative contribution of team members and weight some more heavily than others. For instance, Mathieu et al. (2014) recently proposed an integrated framework of team composition models that address various team compositional mixes across various team performance episodes, which incorporates elements of membership dynamics.

Current approaches to team composition research focus the conceptualization of team composition factors as a property of the team’s constituent *people*, primarily due to the common conceptualization of team composition as a function of team member *attributes* (Bell et al., 2018). In other words, team composition is traditionally based upon the characteristics of the team members themselves and is consistent with the focus on the people element of the definition of teams. However, we have illustrated the practical difficulty and complexity of creating a clean operationalization of team composition in small organizations using this traditional approach. In addition, these more traditional approaches do not account for team members in small organizations who operate as interdisciplinary individuals with multiple attributes relevant to team goals, when team members serve as members of multiple teams, and when team membership is inherently fluid due to the nature of the organizational system and context in which such teams operated.

We propose that for teams in small organizations, a better approach for evaluating team composition may be a focus on “roles and responsibilities”. This approach focuses primarily on the collection of team *roles* necessary for effective team performance, and the subsequent skill variety necessary to fulfill such roles, as opposed to individuals. This new approach to evaluating team composition is especially relevant when working with a fluid team consisting of people who serve in more than one role or represent a variety of skills. Higgins et al. (2012) provided some foundation for this argument by proposing that exploring role stability and searching for team members with capabilities to meet the *role* can help teams address membership changes and mitigate its impacts on team processes performance. Further, Bushe and Chu (2011) offered some practical solutions to overcome individual knowledge loss issues due to team member fluidity that also emphasized the necessity of generalized roles and standardized skill sets. Such a conceptualization would require measurement of skill variety with respect to the collection of roles within the team, as opposed to the properties of the constituent people. Driskell et al. (2017) generated a summary of the team roles literature, proposing 13 primary team role clusters that may provide further insight into the opportunities for utilizing roles as the unit of interest.

The implication of this approach for the study of teams is that we must attend to the issue of what roles individual team members have in the team instead of or in conjunction with the traditional approach. An important consideration when evaluating role, knowledge, and skills is that these are harder to evaluate and measure compared to demographics or job function. Further, in larger organizations, the use of teams composed across departments or job functions is because it is assumed that team members from different departments or job functions will have different knowledge and skills. In small organizations, one individual may represent multiple job functions and/or multiple knowledge and skill domains, leading to increased complexity in how team roles, knowledge and skills are measured.

Based on the discussion above, we provide some suggestions as to how this can be measured and evaluated. First, it is important to understand why specific individuals are needed on a team – what specific knowledge and/or tasks they are performing. However, this is not always an easy or straightforward task. We can ask individuals why they are on the team, what role or task they perform and so on. However, individuals may not always be aware of why they have been asked to be on a specific team, especially in newly formed teams, or those that lack a clearly defined purpose or goal. It is not uncommon to hear individuals say that they are on the team because their manager asked (or told) them to be on the team. Their understanding of the role may further change over time (DeRue and Morgeson, 2007), as the team and team tasks change. While this approach may result in missing information or unclear information, it is still important to understand team composition. This brings to light another concern, which is how to address the issue that team members may not be aware of the purpose behind their assignment to the team. Here it is important to stress two points. This lack of awareness and knowledge may be in and of itself a variable of interest. That is, whether a team member is aware of his or her role on the team may contribute directly to individual and team performance. As such, it is important not to treat this response as missing information or missing data, but rather as a valid data point.

Second, a potential way to address this would be to obtain information from the manager assigned to the team. A difficulty that may arise here is when the manager’s expectations and reasons for assigning someone to a team are not in line with the team member’s perception. It then becomes important to determine whether to use managers’ data or that by team members in conceptualizing roles. Another methodological approach would be to identify a list of roles, knowledge, and skills necessary for the specific team, and then identify which individuals fulfill each of these. This would allow us to identify those interdisciplinary individuals on the team as well as identify which of the roles, knowledge and skills are addressed, and which are missing. Further, it will also allow us to determine whether there is a degree of overlap across individuals in their roles in the team and the knowledge and skills they have. This approach differs substantially from current approaches to team composition, as it focuses on identifying the roles, skills, and knowledge first. This means that the researcher must have a good understanding of the team and its purpose to develop such a list.

#### Multiple Team Membership

While multiple team membership has its benefits, such as increased variety of information available and information exchange, it also has its drawbacks such as costs of information and task overload and challenges with coordination (O’Leary et al., 2011; Pluut et al., 2014). In small organizations, multiple team membership may be a necessity due to limited numbers of personnel with the required skills sets from which to create teams to achieve the goals and objectives of multiple teams. In addition, the use of multi-team systems can require overlap of members across the different teams (O’Leary et al., 2012). Thus, any one employee may act as a member of key teams necessary for the organization’s success and the multi-team system. As mentioned in the prior section, many of these team members may also fit the category of “interdisciplinary individuals” who fulfill various functional expertise and role needs that might have otherwise been filled by more than one person. Thus, the ability to effectively operationalize team composition would require careful tracking of individuals’ membership on the teams of interest and is complicated by the multiple duties and functionalities associated with some, but not all, team members.

One important methodological challenge associated with multi-team membership is non-independence of data (Bliese, 2000). In asking participants for their opinions, whether by survey or interview, the individual in fact represents multiple teams of interest. Team members may be uncertain about their membership in any given team, or may disagree with others about what constitutes team membership (Margolis, 2020). This leads to a number of problems. Suppose we do not specify which unit the individual needs to represent while taking the survey or conducting the interview. In that case, the person may represent one or multiple teams – and we do not necessarily have a way to know that. This issue can be addressed by specifying which unit the person needs to represent – however, that means either not eliciting the perspective representing the other team the person belongs to or responding to multiple surveys resulting in data that are not independent. This problem is then compounded by the fact that the number of participants is small to begin with. Making the decision to remove a participant from one team they belong to would further reduce the number of participants.

#### Fluid Team Membership

The fluidity of individual membership in any given team also presents methodological and practical challenges. In addition to having some expertise available at certain points but not others, the nature of work in some small organizations (e.g., healthcare and fire stations) may naturally include shifts and shift changes. Such events result in frequent changes of team members who assume team roles and engage in team tasks and processes; this fluidity emerges as a result of work schedule changes and staffing fluctuations. Team membership fluidity exists in organizations regardless of size (Bushe and Chu, 2011). However, in small organizations, individual team members are more likely to have previous interactions with other team members. That is, the pool of potential team members means that everyone likely knew most everyone else. In larger organizations, familiarity with other personnel may not be as complete and thus the fluidity for large organizations meant unfamiliarity between members on new teams. In contrast, the greater volume of previous interactions in small organizations may have occurred either in a professional setting (i.e., at work), or in some cases in the broader community (i.e., church and baseball game). As a result, fluidity of teams in small organizations may still exist, but the naturally occurring familiarity between personnel meant that the new team members likely had a greater level of personal history introducing unique team properties.

From a methodological standpoint, the phenomenon of fluidity and its implications may be very different. In larger organizations, fluidity typically implies that members are less familiar and therefore require a period of adjustment after team membership changes. However, this is less likely to be the case in smaller organizations due to pre-existing familiarity. In small organizations, change in team membership may have a stronger effect on team composition. For example, small organizations might only employ a single professional in a given area. If this individual is unable (due to job demands) or unwilling to participate in a team, in effect, there is no ability to “replace” the expertise in-house. In larger organizations, while team composition may change in terms of individuals, it may be easier to ensure that various areas of expertise necessary for team functioning are represented simply due to a greater number of employees with similar functional expertise.

In addition, as noted above, changes in team composition, that is, the replacement of team members, may have different effects in smaller organizations, as team members may already have familiarity with the new incoming member. The research on changing team membership is limited; however, in most cases, the underlying assumption is that the incoming new team member is new to the team and some period of adjustment is required, potentially for both new and existing members. When in fact a new team member is not an “unknown” to the team, it is unclear what effect this may have on the team and team processes. Thus, the effects of team fluidity and changing team membership may be different in smaller organizations because of relative familiarity. However, at this point, research on team fluidity and membership change for the most part has not addressed why these may influence the outcome, and what role familiarity plays in this process.

Therefore, the phenomenon of interest must be more specifically defined and measured. We proposed that team members’ familiarity with one another and previous history together must be measured directly. That is, just because a team member has changed, does not necessarily mean that the team requires a period of adjustment. In fact, some or all of the team members may have worked with this person before. Therefore, a more direct measure of whether the team, and individual team members have worked with the new team member, may be a more fruitful approach. For example, social network analysis may be a useful tool to identify degree of familiarity between team members including during change to team membership. That approach however, brings another issue to fore. At what point do we argue that the “team” is familiar with that person? How many of the team members or what proportion needs to have that familiarity?

Over time, teams may also experience member fluidity due to individuals leaving the organization (i.e., employee turnover), competing priorities (e.g., the individual’s primary role responsibilities took precedence over regular participation in the team), and in some cases, lack of engagement even when time was allowed for members to participate. Thus, specific personnel who participate in teams may change over time, though in some cases the desired or required expertise and functional roles for team membership remain the same. Such fluidity in staffing may also affect team membership and participation in *ad-hoc* teams that form to address time limited events, such as post-event debriefs. In such cases, only those working and available at the time of the event would have participated in these teams. Tracking individuals who participate in these team from organizational records can provide some evidence of membership; however, the issues described in the section above related to team composition make this approach incomplete.

These fluidity issues make it difficult for both researchers and team members to clearly identify and operationalize some of the fundamental properties of teams as would be expected and characterized under traditional working definitions of what it means to be a “team.” Methodologically, this indicates that measuring team composition at one specific point in time may not accurately reflect the team composition across the duration of the team’s life cycle. Further, it is unclear whether the different reasons for composition change will influence the composition itself, team processes that emerge as a result of the composition of the team, or the team’s outcomes. Similar to our recommendation regarding team composition, it is important to evaluate team fluidity and membership change from the standpoint of the specific individual (new person) and how fluidity addresses the role and expertise needs of the team.

### Core Processes and Emergent States

In this section we discuss unique methodological challenges for measuring and evaluating team processes and emergent states in small complex organizations. We focus on challenges related to evaluating communication, coordination, and coaching. For each of these challenges we draw on our theoretical discussion outlined in the previous sections, particularly when considered in light of the team composition issues described in the prior section, and then offer some potential solutions. Table 3 provides a summary of our discussion of challenges and proposed solutions.

**Table 3:** Team process methodological considerations for teams in small organizations.

Team process factor	Process factor defined	Special considerations for study in small organizations	Methodological and evaluation considerations	Potential solutions
Coordination	Interaction processes that allow for integration of collective tasks (Okhuysen and Bechky, 2009)	Team members who wear multiple hats in single roles may increase complexity of activity and task coordination	Multiple team membership may result in overrepresentation of individuals under traditional methods of measuring connection and interaction Team composition complexities necessitate rich descriptions of team processes in action and over time Multiple team membership creates challenges to clearly identifying direct and indirect influences of communication within and across teams	Identify both formal and informal roles and clarify expected vs. actual completion of tasks associated with each to inform action processes needed for team performance Weigh responses based on team member identification when the person belongs to more than one team Establish interaction profiles to clearly characterize processes that emerge within and across teams Reword measures related to hierarchy and leadership to more appropriately apply to flatter (i.e., smaller) organizations Weight team interactions by member profiles to statistically control for compositional effects that may bias estimates of processes
Boundary spanning	Establishing and managing interactions with others external to the team to meet team goals (Van Osch and Steinfield, 2016)	Multiple teams with compositional overlap offers opportunities for natural networking and connection between teams Access to unique ideas and resources may be constrained by frequent overlap of team members		
Coaching (leadership)	“Ability to influence, motivate, and enable others to contribute to the effectiveness and success of the organizations of which they are members” (House et al., 2004, p. 15).	Potential for fewer levels of management and leadership Leadership may be more involved and aware of day-to-day activities and needs Greater knowledge of organizational needs offers opportunity for more direct influence on team processes and activities		
Communication	Dialogue, conversations, and meetings that occur between members during regular operations	Overlap in team membership and lack of diversity creates homogeneity in team communication and interaction patterns		

#### Communication

For our purposes, team communication refers to the dialogue, conversations, and team meetings between team members during regular operations. The methodological issue that arises here stems in part from the compositional issues previously discussed. Specifically, recent research indicated that individuals have tendencies toward certain types of behavior within team interaction (Lehmann-Willenbrock et al., 2015). A person may have an interaction profile that indicates the kinds of behavior they are most prone to when interacting with others in a group or team setting (Lehmann-Willenbrock et al., 2015). Given the issues of fluidity and diversity in teams in small organizations, a given person could be part of several teams and share their thoughts and opinions across the teams. They may do so in their unique profile pattern of behavior and perhaps exhort a disproportionate influence on team and organizational activities compared to more unique non-overlapping teams that occur in large organizations. For example, suppose a given person believed that a certain solution worked to solve a problem in the small organization and then shared this in each of the teams they are involved with. In that case, their solution will be heard by more than a person who is a member of just one team. Alternatively, from an interaction profile perspective, a person who is prone to complaining behavior would likely do so in all the teams they are a part of thereby creating the potential for complaining cycles that are known to derail and hamper team performance (Lehmann-Willenbrock et al., 2011). Assuming we follow current preferred processes for coding the behavior in these team interactions (Lehmann-Willenbrock and Allen, 2018), the coded data will be skewed toward the individuals who are found across the teams and lead to conclusions that are different and inconsistent with findings in larger organizations with non-overlapping group membership.

Given this problem, there are a few potential solutions for consideration. First, simple awareness of this compositional issue that manifests itself in the processes can help with how conclusions are drawn. Specifically, researchers can simply acknowledge that small organizations have a compositional issue that makes interaction different and that conclusions drawn only apply to similar small organizational contexts. In this case, generalizability is limited, but the findings are no less meaningful for the many small organizations that exist. Second, if the desire is to more closely generalize to other groups and teams, then another approach may be to weight the interactions by profile regardless of organizational size. Since all participants likely have a particular profile, identifying each person’s profile and then weighting their contributions for any analysis involving those who occupy multiple teams could provide a way to statistically control for this compositional process bias (Lehmann-Willenbrock and Allen, 2018).

Our discussion also suggested challenges in measuring team member engagement in boundary spanning (Marrone, 2010). Specifically, if the team composition is overlapping across the boundary spanning teams, some individuals are overrepresented in collecting measures of engagement in boundary-spanning across teams. Potential solutions to this challenge are the weighting of responses based on the relative level of team member identification with a team; however, this also requires that team members overcome team identity challenges associated with multiple team membership. Alternatively, if the teams are adequately large, computations of the homogeneity of responding could indicate the need or lack of need of the overlapping individuals. For example, interclass correlations (ICCs), rwg’s, and other measures of response homogeneity could be computed with and without the overlapping individuals. Upon comparison, their inclusion or exclusion from the subsequent analysis may be considered. However, due to the smaller number of individuals in the organization, excluding individuals from teams can be problematic by underrepresenting the number and possible degree of boundary spanning that occurs. Another potential solution could be using social network analysis to identify individuals that serve as either formal or informal boundary spanners.

#### Coordination

Formal role definition and task division within teams in small organizations may not be strictly adhered to for a variety of reasons. First, as noted in the previous section, some individuals may serve in multiple roles due to the organization’s small nature. Given the job demands of holding multiple roles this requires a degree of flexibility in how work tasks are coordinated, and what aspects of the role each individual assumes at any given point in time. Second, certain tasks may be viewed as “everyone’s responsibility” which creates a diffusion of responsibility across members of a variety of teams. This diffusion of responsibility across multiple actors makes it more difficult to ascertain who was responsible for a particular task. This diffusion across roles, and the emergence of informal roles creates difficulties to assess roles in teams in the traditional sense using more traditional approaches. Other complicating factors emerge due to fluid team membership and multiple team membership. As noted, because of the organization’s small size, these individuals may have served a function of coordination in an unofficial capacity.

One methodological approach to address this issue is to directly ask employees to indicate who was responsible for performing any one task. This methodological approach may be more time consuming for both researchers and participants, as it is more granular. However, it will result in a more accurate reflection of task division and subsequent coordination necessary to complete tasks. A second approach is to utilize activity traces to understand the actual completion of tasks. This may include reviewing records of pages, entries into various workflow programs (e.g., electronic medical records and project management tools), and emails (Rosen et al., 2015). Workflow mapping may also help to understand various tasks and players involved. At the same time, one must be mindful of the formal and informal roles that individuals have in the organization, and how such roles influence the distribution and completion of work tasks. The formal roles often define the team’s composition, as noted previously, whereas the informal roles and tasks are essential for team processes and coordination.

#### Coaching

One methodological challenge to capture leadership processes in teams within small organizations is that measures previously used in larger settings may have language that is not clear to the respondents due to the different nature of the leadership hierarchy, and may not include items focusing on the relevant levels of leadership in a particular context that may influence the processes and outcomes of interest. Modification of survey items may be necessary to address this issue. That, however, may require additional checks on reliability and validity.

In addition, the hierarchical nature of the leadership structure common to larger organizations results in a more directive leadership especially from top management (Oshagbemi and Gill, 2004). An important implication of this finding is that the top management team (TMT) of small organizations is likely more accessible to employees for discussion, suggestions, and conversations, and thus may have a greater direct and indirect impact on individual, team, and organizational performance. From a methodological perspective, the TMT in smaller organizations may be more accessible to the researchers as well. These circumstances may enable researchers to utilize brief interviews with TMT members to capture a rich qualitative description of top leadership understanding and perceptions (which is uncommon), and improve the ability to characterize the impact of leadership processes on team composition, processes, and outcomes. However, these methods remain limited in their ability to accurately capture leadership dynamics and their effects over time.

### General Methodological Challenges and Solutions

Below are two specific methodological issues that affect the study of teams in small organizations that are common or related to both input and process issues. Table 4 provides a summary of these recommendations.

**Table 4:** General methodological considerations for studying teams in small organizations.

Overarching methodological problem	Special considerations for study in small organizations	Potential solutions
Measurement	Context of small organizations may result in fewer barriers to implementing technologies necessary to measure team processes and dynamics as they emerge in real time	Implementation of alternative unobtrusive data collection methods, such as sensor-based activity trace mechanisms and audio/video, to explore interactions and team dynamics as they emerge over time
Small sample size	Organizational size places natural constraints on the possible number of teams and multi-team systems available for study Multiple team membership considerations further constrains the number of distinct teams within the organization	Engagement of multiple organizations to enhance sample size and potential for generalizability Utilize longitudinal study designs with data collection at multiple time points Mixed methods and triangulation of data points from multiple sources increases depth of understanding team phenomena

#### Measurement

Traditionally, team processes have been measured using self-reported questionnaires and treated as static constructs (Kozlowski and Chao, 2012), often due to the relative ease of data collection using such methods. However, researchers have readily acknowledged that team processes are dynamic (Marks et al., 2001; Kozlowski, 2015), but such dynamics are often missing in how team researchers conceptualize team processes (Cronin et al., 2011; Cronin, 2015). New directions in the conceptualization and measurement of team processes have been proposed and explored recently in the literature (e.g., Kozlowski, 2015; Kozlowski and Chao, 2018; Lehmann-Willenbrock and Allen, 2018). These approaches build upon traditional methods by which team processes are captured and framed as “frozen” mediating actions (Kozlowski, 2015) and enable evaluation of team dynamics as processes are so often conceptualized. For instance, Kozlowski (2015) discussed opportunities to use new and emerging methods to collect data, such as team interaction sensors and computational modeling (Kozlowski and Chao, 2018), and to enhance the use of existing methods, such as highly descriptive qualitative approaches including observations, interviews, and document analysis. Rosen et al. (2015) proposed various sensor-based measurement and activity trace mechanisms that could be used to capture teamwork and team processes in action. Such options include the use of RFID tags, video and audio recording devices, paging systems, and entries into electronic medical record systems.

Methods that enable rich descriptions of the team processes in action, and over time are needed to address many of the challenges we discussed above (Lehmann-Willenbrock and Allen, 2018). One important factor for consideration when studying small complex organizations is that this context may provide one of the best testing grounds for these approaches to studying team processes. Because the organization is relatively small, it may be easier to conduct this type of dynamic research where audio or video recording may be needed or other sensor-oriented devices must be deployed to track team processes as they emerge over time. The practical issues of managing people in teams, and managing equipment and data, may be easier to implement in such organizations. Since these small organizations have fewer layers in their hierarchy, it may be less difficult to obtain organizational approval for conducting such research. Further, using alternative data sources (e.g., audio, video, or sensors) may be more manageable due to the smaller size. In situations where real-time monitoring of team processes lacks feasibility, other less obvious sources of data such as event reports and data repositories may shed light on team activities.

#### The Small N Problem

Another important methodological implication has to do with the number of teams available for study in small organizations. When studying teams, teams are likely the unit of analysis (Forsyth, 2018). As such, it is important that we have a sufficient number of teams to allow us to analyze team data and reach conclusions based on statistical evidence. When dealing with smaller organizations, the number of teams available for study can be relatively small, depending on the organization’s size and structure. A related problem, specific to highly complex organizations is the existence of multi-team systems (MTS). While multiple MTS may be available for study in larger organizations, smaller organizations will have far fewer or only a single MTS. To augment the number of teams or MTS, we must include multiple organizations. This has two positive outcomes. First, sample size is increased, leading to increased power to detect effects that may exist. The second is that we create greater opportunity for generalizability. The concern is the potential for various uncontrolled and influential variables that differ from one organization to another, and may influence the results of such studies (e.g., organizational culture). In addition, as can be seen in the four studies that have evaluated small organizations (Table 1), all have opted to include multiple organizations, and most (three out of the four) included organizations across a wide variety of industries. While this strategy improves sample size and generalization, industry level nuances may be lost.

Combining data across multiple organizations is not always sufficient to deal with the sample size issue at the MTS or team unit of analysis. This issue is particularly challenging when the primary outcome of interest resides at a level greater than the unit of the team or MTS, such as the organization. Such small sample sizes place limits on the statistics available to evaluate the effects of MTS on organizational outcomes using quantitative methods. More complex methodologies that take into account nested data such as HLM cannot be conducted with such a small data set. The research literature in I/O and Management often does not address quantitative solutions for using small N. However, there are some solutions available in domains such as clinical Psychology or school Psychology as well as researchers studying extreme groups such as military and NASA teams, which we may be able to adapt for our needs. For example, one approach recommended for testing improvement when you have small sample (including a sample of 1), is incorporating multiple baseline data points and follow-up (post intervention) data points and evaluating the trend. Further, the practical challenges of working with multiple MTSs in real world settings presents the opportunity to step back from our traditional quantitative focus, and utilize qualitative methods to provide rich descriptions that fully characterize the compositional elements of MTSs. Another important way to address this issue of small N is that of triangulation, for example combining data from both qualitative and quantitative data collection. For example, NASA is very interested in the effects of long-term space flight on astronauts’ physical and psychological well-being and often collect quantitative and qualitative data using simulations with flight teams. While addressing small N is not unique to teams research and has been addressed in other domains (as noted above), this is still a critical issue that must be tackled. Not conducting research within these organizations means that we are ignoring not only an important contextual factor, but also a large proportion of employees and teams in the workforce. Finally, it is important to combine and identify research from other sources in which a small number of teams can be studied. For example, interest in the study of teams for long-duration space flight resulted in NASA researchers focusing on the study of teams in extreme conditions, or analog teams (Landon et al., 2018). Similarly, research on small complex organizations can evaluate potential analogs, or similar organizations, that may allow for the increases in sample size.

## Conclusion

Current team research has acknowledged that context can be critical to our understanding of team effectiveness (Bell et al., 2018; Golden et al., 2018). The purpose of this paper was to identify some of the differences between teams in larger compared to smaller organizations, and pinpoint potential methodological challenges associated with conducting team research in such organizations. We believe that it is critical that our understanding of teams, teamwork, and MTS apply to small organizations; however, rigorous testing and application of our existing theories and findings to this unique setting are necessary to fully determine whether findings are universal or more specific to the context of larger organizations or highly unique settings.

The issues, challenges, opportunities, as well as potential solutions raised apply to a variety of small organizations such as small businesses and the ever-growing start-up company industry. Further, there are likely many more issues, challenges, and opportunities that will emerge in these other types of small organizational settings that will inform our understanding of team composition and processes and illuminate additional methodological issues that affect the study of teams. This paper should serve to facilitate both conversation about and activity to advance the study of teams in small businesses and MTSs in smaller organizations.

## Author Contributions

All authors listed have made a substantial, direct and intellectual contribution to the work, and approved it for publication.

### Conflict of Interest

The authors declare that the research was conducted in the absence of any commercial or financial relationships that could be construed as a potential conflict of interest.
